# A Mouse Model with Ablated Asparaginase and Isoaspartyl Peptidase 1 (*Asrgl1*) Develops Early Onset Retinal Degeneration (RD) Recapitulating the Human Phenotype

**DOI:** 10.3390/genes13081461

**Published:** 2022-08-17

**Authors:** Pooja Biswas, Anne Marie Berry, Qais Zawaydeh, Dirk-Uwe G. Bartsch, Pongali B. Raghavendra, J. Fielding Hejtmancik, Naheed W. Khan, S. Amer Riazuddin, Radha Ayyagari

**Affiliations:** 1 Shiley Eye Institute, University of California San Diego, La Jolla, CA 92093, USA; 2 School of Biotechnology, REVA University, Bengaluru 560064, India; 3 National Institute of Biomedical Genomics, Kalyani 741251, India; 4 National Eye Institute, National Institute of Health, Bethesda, MD 20892, USA; 5 Ophthalmology and Visual Sciences, University of Michigan Health, Michigan Medicine, Ann Arbor, MI 48109, USA; 6 Wilmer Eye Institute, Johns Hopkins University School of Medicine, Baltimore, MD 20817, USA

**Keywords:** *ASRGL1*, animal model, CRISPR/Cas9, early onset degeneration

## Abstract

We previously identified a homozygous G178R mutation in human *ASRGL1* (*hASRGL1*) through whole-exome analysis responsible for early onset retinal degeneration (RD) in patients with cone–rod dystrophy. The mutant G178R ASRGL1 expressed in Cos-7 cells showed altered localization, while the mutant ASRGL1 in *E. coli* lacked the autocatalytic activity needed to generate the active protein. To evaluate the effect of impaired ASRGL1 function on the retina in vivo, we generated a mouse model with c.578_579insAGAAA (NM_001083926.2) mutation (*Asrgl1^mut/mut^*) through the CRISPR/Cas9 methodology. The expression of ASGRL1 and its asparaginase activity were undetectable in the retina of *Asrgl1^mut/mut^* mice. The ophthalmic evaluation of *Asrgl1^mut/mut^* mice showed a significant and progressive decrease in scotopic electroretinographic (ERG) response observed at an early age of 3 months followed by a decrease in photopic response around 5 months compared with age-matched wildtype mice. Immunostaining and RT-PCR analyses with rod and cone cell markers revealed a loss of cone outer segments and a significant decrease in the expression of *Rhodopsin*, *Opn1sw*, and *Opn1mw* at 3 months in *Asrgl1^mut/mut^* mice compared with age-matched wildtype mice. Importantly, the retinal phenotype of *Asrgl1^mut/mut^* mice is consistent with the phenotype observed in patients harboring the G178R mutation in *ASRGL1* confirming a critical role of ASRGL1 in the retina and the contribution of *ASRGL1* mutations in retinal degeneration.

## 1. Introduction

Inherited retinal dystrophies (IRDs) result in irreversible loss of vision due to the progressive degeneration of the retina. IRDs are considered genetically heterogeneous. There are over 280 genes involved in retinal degeneration, which can present with autosomal dominant, autosomal recessive, X-linked, mitochondrial, and complex modes of inheritance [1,2,3,4,5] (https://sph.uth.edu/retnet/home.htm accessed on 1 January 2022). Mouse models have been instrumental in elucidating the mechanisms by which these important genes lead to pathology [2]. A large number of mouse models of inherited retinal degeneration have been developed by introducing mutations in IRD-associated genes, and several naturally occurring mouse models with retinal pathology have also been characterized [6,7,8,9,10]. In this paper, we describe a novel mouse model with a loss of ASRGL1 expression caused by a c.578_579insAGAAA (NM_001083926.2) mutation introduced by editing the genome using CRISPR/Cas9.

A homozygous missense mutation G178R in the human asparaginase like-1 gene (*ASRGL1*) is associated with early onset retinal degeneration in a consanguineous Pakistani pedigree [11]. *ASRGL1* encodes an enzyme that is synthesized as an inactive precursor protein that becomes active after undergoing autocatalytic intramolecular processing, with the active form able to catalyze the hydrolysis of L-asparagine and isoaspartyl-peptides [11,12]. Expression studies revealed that there are high levels of *ASRGL1* in the optic nerve, retina, heart, and brain, with low to minimal levels in all other tissues in mice [11]. ASRGL1 localizes to the photoreceptor layer and is known to be highly expressed in the retina at older ages, giving it a putative role in the maintenance of aging retinal tissue. The G178R mutation detected in patients with early onset IRD affects the autocatalytic activity of ASRGL1 [11]. Despite this progress, the molecular mechanism underlying ASRGL1’s involvement in retinal degeneration is unknown. In this study, we showed that a mouse model with a homozygous null allele provides key insights into the role of *ASRGL1* in retinal degeneration.

## 2. Methodology

**Figure 1:**
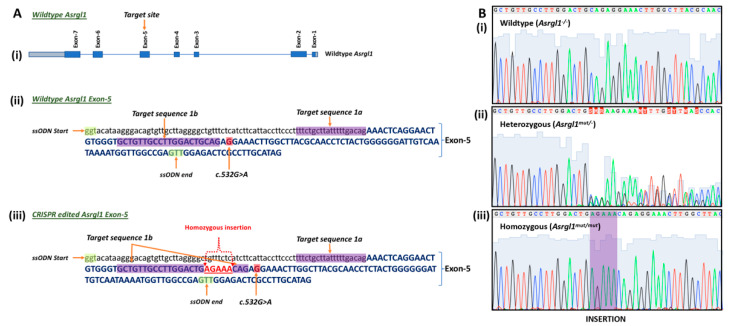
**Design of the *Asrgl1^mut/mut^* mouse model:** (**A**) Schematic diagram showing (**i**) target site of *Asrgl1* gene and (**ii**) complete sequence of exon-5 of wildtype and (**iii**) complete sequence of exon-5 *Asrgl1^mut/mut^* mouse model with the c.578_579insAGAAA. (**B**) Electropherogram showing the selected sequence of (**i**) wildtype *Asrgl1*, (**ii**) with 5 base insertion mutation c.578_579insAGAAA in heterozygous and (**iii**) homozygous states.

***Generation of Asrgl1^mut/mut^ mouse model using CRISPR/Cas9 system:*** The *Asrgl1* gene ablated mouse model (*Asrgl1^mut/mut^*) was generated under the authorization of the Institutional Animal Care and Use Committee (IACUC) of the University of California San Diego using a CRISPR/Cas9 system (Figure 1A) [13,14]. For genome editing, a concentrated 25 μL cocktail of 100 ng/μL single-stranded oligodeoxynucleotides (ssODNs) as a repair template was used along with 20 ng/μL sgRNAs and 50 ng/μL Cas9 mRNA. The reverse complementary ssODNs sequence is shown as follows: 5′—(~60—nt 5′ homology) CCAACTCGGCCAACCATTTTATTGACAATCCCCCCAGTAGAGGTTGCGTAAGCCAAGTTTCTTCTGCAGTCCAAGGCAACAGCACCCACAGTTCCTGAGTTTctgtcaaaaataagcagaaaagggaaggtaatgaagatgagaaacagcccctaagcaacactgtcccttatgtacc (~60—nt 3′ homology)—3′. This CRISPR/Cas9 cocktail was microinjected into the embryos of C57BL/6 mice.

**Figure 2:**
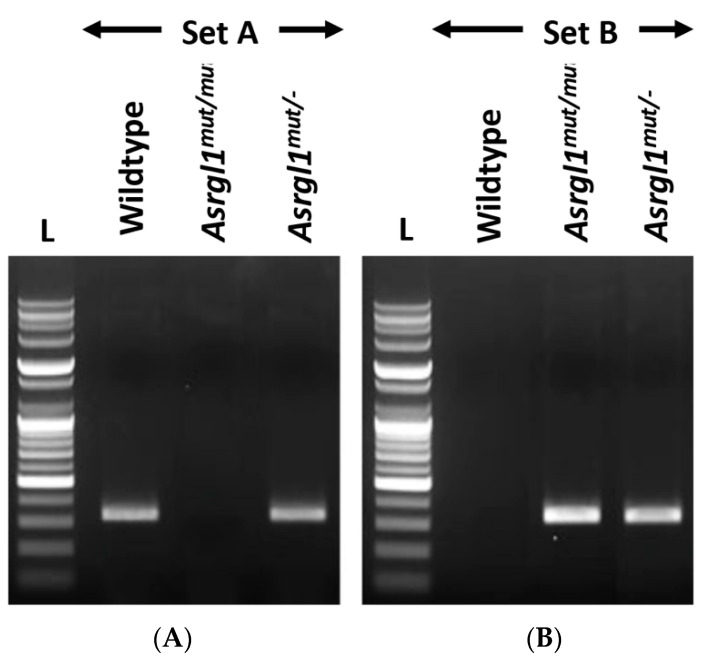
**Gel electrophoresis of the *Asrgl1^mut/mut^* mouse:** (**A**) Set A primers amplified a 328 bp product without the 5 base insertion at c.578 position from wildtype and heterozygous *Asrgl1^mut/−^* alleles. (**B**) Set B primers amplified a 333 bp product from the homozygous and heterozygous *Asrgl1^mut/−^* alleles.

**Genotyping *Asrgl1 ^mut/mut^* mice:** Three unique primers located in the region flanking the mutation or insertion site were generated to amplify either the wildtype or mutant *Asrgl1* allele to determine the genotype of *Asrgl1^mut/mut^* mice (Figure 2, Table 1). Amplification reaction with the forward primer “mAsrgl1 wt F” and the reverse primer “mAsrgl1 uni R”, indicated as primer set A, generated a 328 bp PCR product of the wildtype allele (Table 2), whereas amplification of the *Asrgl1^mut/mut^* allele with primer set B (“mAsrgl1 mut F” and “mAsrgl1 uni R”) generated a 333 bp product. Sanger sequencing of these amplification products enabled the detection of the specific sequence alteration to confirm the genotype (Figure 1B and Figure 2). These mice were also genotyped for *Rd1* and *Rd8* as previously described [15].

***Ophthalmic evaluation by in vivo imaging:*** Mice at desired ages (3, 5, and 10 months) were anesthetized with a mixture of ketamine (93 mg/kg body weight) and xylazine (8 mg/kg body weight) intraperitoneally administered following the UCSD’s Controlled Substances Use Authorization (CSUA). During this procedure, mice were kept on a heating pad at 37 °C to maintain normal body temperature. Prior to imaging, the eyes were dilated with topical proparacaine hydrochloride (0.5%) (NDC: 17478-263-12, Akorn, Inc. Lake Forest, IL, USA) and topical tropicamide (0.5%) (NDC 17478-101-12, Akorn, Inc. Lake Forest, IL, USA), followed by phenylephrine (2.5%) (NDC: 17478-201-15, Akorn, Inc. Lake Forest, IL, USA). For imaging, 3-, 5-, and 10-month-old *Asrgl1^mut/mut^* mice and 10-month-old wildtype control mice were used. Six mice of each genotype were used for ophthalmic evaluation at each age point.

A Spectralis™ HRA + OCT machine (Heidelberg Engineering Inc., Heidelberg, Germany) was used for scanning laser ophthalmoscopy, infrared, and spectral domain optical coherence tomography imaging. To improve imaging, a custom-made sterilized PMMA contact lens for mice was used to keep eyes moist and lubricated in order to elude cataractogenesis caused by drying of the surface of the eye. To measure retinal thickness, Heidelberg eye explorer software (Heidelberg Engineering, Inc. Heidelberg, Germany) was used. Three-, five-, and ten-month-old *Asrgl1^mut/mut^* mice were compared with age-matched wildtype controls to study retinal changes by using fundoscopy and fundus autofluorescence.

***Immunohistochemistry:*** Cryosections of 3-, 9-, 13-, and 17-month-old *Asrgl1^mut/mut^* and 12-month-old wildtype C57BL/6 mouse eye were used to perform immunohistochemistry as previously described [15]. Rabbit anti-ASRGL1 antibodies (1:100) (ProteinTech Cat-11400-1-AP), anti-rabbit polyclonal OPN1MW (1:200) (AB5405, Millipore Sigma, Burlington, MA, USA), anti-goat polyclonal OPN1SW (1:200) (Santa Cruz Biotechnology, Dallas, Texas), anti-mouse rhodopsin monoclonal antibody (1:200) (MA1-722, Thermofisher Scientific, Carlsbad, CA, USA), anti-mouse ATP1A1 monoclonal antibody (1: 200) (M7-PB-E9, Thermofisher Scientific, Carlsbad, CA, USA), AlexaFluor-488-conjugated donkey anti-rabbit secondary antibody (1:2000) (Invitrogen, Carlsbad, CA, USA), AlexaFluor-555-conjugated donkey anti-goat secondary antibody (1:3000) (Invitrogen, Carlsbad, CA, USA), and AlexaFluor-555-conjugated donkey anti-mouse secondary antibody (1:3000) (Invitrogen, Carlsbad, CA, USA) were utilized for staining. The nuclei were stained using a Vecta shield DAPI medium (H-1500-10, Vector Laboratories, San Diego, CA, USA). Images were captured using a Nikon confocal microscope system (A1R STORM, Nikon; Melville, NY 11747, USA) at 60× objective lens (0.85 numerical aperture) and processed using Adobe Photoshop CS6 (Adobe Associates, Inc. Santa Rosa, California).

***Quantitative transcript expression:*** The retinal layer was dissected from 3- and 12-month-old *Asrgl1^mut/mut^* and 12-month-old wildtype control mice. Isolation of retinal RNA followed by reverse-transcription reaction and calculation of *Opn1mw*, *Opn1sw*, and *Rhodopsin* expression relative to the housekeeping genes *Gapdh* and *Actb* were performed as described earlier [16].

***Quantification of asparaginase activity:*** Asparaginase enzymatic activity of the 11- to 15-month-old *Asrgl1^mut/mut^* and 10-month-old wildtype control mice was measured in freshly dissected retinal tissue lysates using an Asparaginase Activity Assay Kit (ab107922, Abcam, Cambridge, MA, USA).

## 3. Results

### 3.1. Generation of Asrgl1^mut/mut^ Mouse Model

The newborn pups were screened for *Asrgl1* sequence changes, which revealed the homozygous insertion of five base pairs AGAAA after amino acid c.578 (c.578_579insAGAAA). Additional genotyping for the *rd8* and *rd1* mutations revealed the presence of the *rd8* mutation in addition to the *Asrgl1* 5 bp insertion (c.578_579insAGAAA), but not the *rd1* mutation. The *Asrgl1^mut/mut^* mice with the *rd8* allele were bred with wildtype C57BL/6 to generate *rd8*-mutation-free *Asrgl1* mutant mice (Figure 1 and Figure 2), and, subsequently, these mice were bred to generate homozygous *Asrgl1^mut/mut^* mice that were free of the *Rd1* or *Rd8* mutations for further studies.

### 3.2. Ophthalmic Evaluation

**Figure 3:**
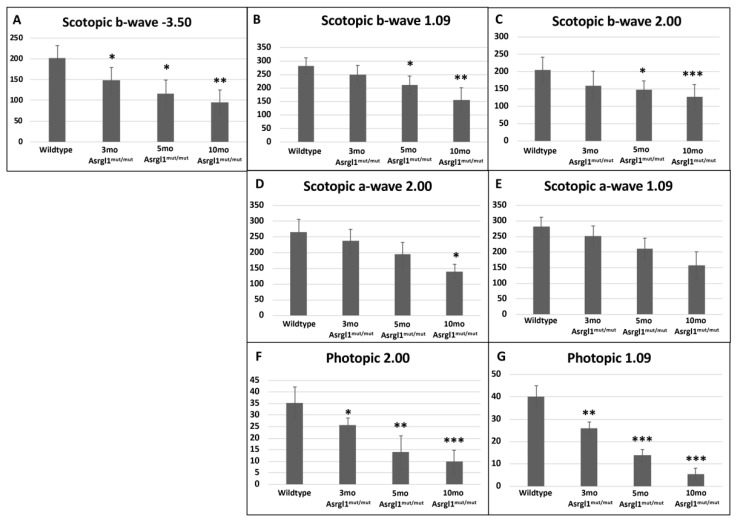
**Full-field electroretinography (ffERG) responses in *Asrgl1^mut/mut^* mice model:** Average scotopic and photopic ERG a- and b-wave amplitudes for 10-month-old wildtype control mice compared with 3-, 5-, and 10-month-old *Asrgl1^mut/mut^* mice at −3.50 log cd.s/m^2^, 1.09 log cd.s/m^2^, and 2.00 log cd.s/m^2^ stimulation intensities. Vertical lines on the bar graphs represent standard error mean. The *p* values < 0.05, 0.01, and 0.001 are indicated with *, **, and ***, respectively.

***Full-field electroretinography (ffERG):*** The retinal function of *Asrgl1^mut/mut^* mice was evaluated by performing an ffERG test at ages 3-, 5-, and 10-months, and the results were compared with those of 10-month-old wildtype control mice. A progressive decrease in mean scotopic b-wave response was noted in *Asrgl1^mut/mut^* mice with age (Figure 3A–C). At 3 months of age, the mean scotopic b-wave response of *Asrgl1^mut/mut^* mice was significantly lower than that of wildtype mice at −3.50 log cd-s/m^2^ (*p* = 0.035), but not at higher intensities (1.09 log cd-s/m^2^ and 2.0 log cd-s/m^2^, *p* > 0.05 for both intensities). However, the mean scotopic b-wave response was significantly reduced in *Asrgl1^mut/mut^* mice compared with wildtype at 5 months (*p* = 0.0048 at log −3.50 cd-s/m^2^, *p* = 0.0217 at log 1.09 cd-s/m^2^, and *p* = 0.0465 at log 2.00 cd-s/m^2^) and at 10 months (*p* = 0.0012 at −3.50 log cd-s/m^2^, *p* = 0.0015 at 1.09 log cd-s/m^2^, and *p* = 0.0008 at 2.00 log cd-s/m^2^) (Figure 3A–C).

Furthermore, the mean scotopic a-wave response of 10-month-old *Asrgl1^mut/mut^* mice was significantly lower compared with the mean response observed in 5-month-old *Asrgl1^mut/mut^* mice (*p* = 0.0123 at 1.09 log cd-s/m^2^ and *p* = 0.0154 at 2.00 log cd-s/m^2^) (Figure 3D,E). Although the scotopic a-wave response was reduced in 3- and 5-month-old *Asrgl1^mut/mut^* mice compared with 10-month-old wildtype mice for 1.09 log cd-s/m^2^ and log 2.00 cd-s/m^2^, the difference in mean amplitude was not statistically significant.

The photopic responses also showed an age-dependent decrease and were significantly reduced in 10-month-old *Asrgl1^mut/mut^* mice compared with wildtype mice (*p* < 0.0001 at 1.09 log cd-s/m^2^ and *p* = 0.0004 at 2.00 log cd-s/m^2^) (Figure 3F,G). Both 3- and 5-month-old *Asrgl1^mut/mut^* mice also demonstrated significantly reduced photopic responses compared with 10-month-old wildtype mice (*p* = 0.0011 at log 1.09 cd-s/m^2^ and *p* = 0.0383 log 2.00 cd-s/m^2^ for 3-month-old mice; *p* = 0.0007 at log 1.09 cd-s/m^2^ and *p* = 0.0056 log 2.00 cd-s/m^2^ for 5-month-old mice). Overall, these results show a gradual but significant decrease in ERG response under photopic conditions with age starting as early as 3 months in *Asrgl1^mut/mut^* mice.

***Fundoscopy:*** Fundus appearance showed no significant difference between the *Asrgl1^mut/mut^* and age-matched wildtype control mice, including the number of autofluorescent spots (Figure S1).

**Figure 4:**
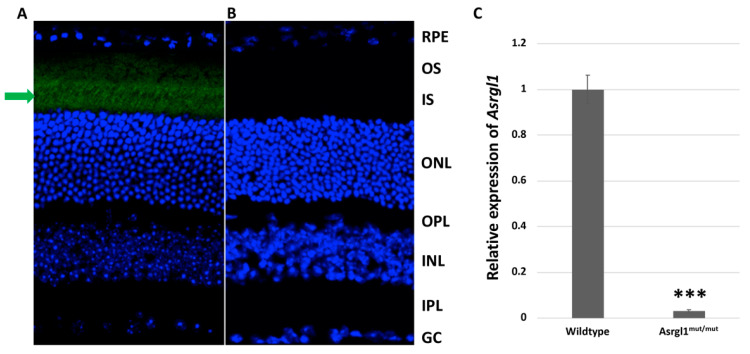
**Expression profile of ASRLG1 in *Asrgl1^mut/mu^*^t^ mouse retina.** (**A**) Immunostaining of retinal sections of 8-month-old wildtype mice detected positive staining of ASRGL1 in the photoreceptor layer. (**B**) Age-matched *Asrgl1^mut/mut^* mice did not show staining of ASRGL1 in the photoreceptor layer. ASRGL1 (green) is stained with specific antibodies, and nuclei are stained with DAPI (blue). RPE: retinal pigment epithelium; OS: outer segments; ONL: outer nuclear layer; OPL: outer plexiform layer; INL: inner nuclear layer; IPL: inner plexiform layer; GC: ganglion cell layer. (**C**) Bar diagram showing *Asrgl1* transcript levels in retinal sections of 3-month-old *Asrgl1^mut/mut^* mutant and wildtype mice. The *p* value < 0.001 is indicated with ***.

***Expression of* ASRGL1 *in the retina of Asrgl1^mut/mut^ mice:*** ASRGL1 localized to the photoreceptor inner segment region of the retina in 8-month-old wildtype mice as shown by the results of immunohistochemistry (Figure 4A). In contrast, immunopositive staining was undetectable in the retinal sections of age-matched *Asrgl1^mut/mut^* mutant mice (Figure 4B), suggesting the absence of protein that is detected by the ASRGL1 antibodies. Consistent with these findings, the level of expression of the *Asrgl1* transcript was also observed to be significantly lower (*p* < 0.0001) in 3-month-old *Asrgl1^mut/mut^* mutant mice compared with that in age-matched wildtype controls (Figure 4C).

**Figure 5:**
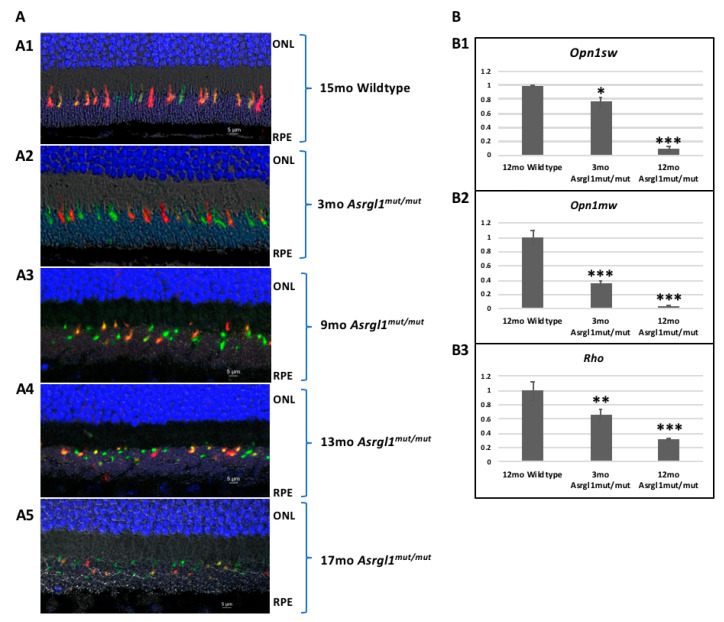
**Expression of photoreceptor markers in *Asrgl1^mut/mut^* mice:** (**A**) **Immunostaining of photoreceptor markers in *Asrgl1^mut/mut^* mice:** retinal sections were stained with OPN1MW/M-opsin (green) and OPN1SW/S-opsin (red). Nuclei were stained with DAPI. (**A1**) Fifteen-month-old wildtype mice. Retina of (**A2**) 3-, (**A3**) 9-, (**A4**) 13-, and (**A5**) 17-month-old *Asrgl1^mut/mut^* mice. Outer segments of OPN1MW and OPN1SW expressing cones are severely abnormal in *Asrgl1^mut/mut^* mice. (**B**) **Levels of expression of photoreceptor marker genes in *Asrgl1^mut/mut^* mice:** levels of retinal cell marker transcripts were measured by qRT PCR in 3- and 12-month-old *Asrgl1^mut/mut^* mice and 12-month-old wildtype mice retinal tissue. (**B1**) Significant decrease in the expression of *Opn1sw* (*p* = 0.0008 for 12 months and *p* = 0.0165 for 3 months) was observed in *Asrgl1^mut/mut^* mice compared with wildtype controls. The *p* values < 0.05, and 0.001 are indicated with *, and ***, respectively. (**B2**) Expression of *Opn1mw* is significantly lower (*p* < 0.0001 for 12 months and *p* = 0.0004 for 3 months) in *Asrgl1^mut/mut^* mice compared with that in wildtype controls. The *p* value <0.001 is indicated with ***. (**B3**) Significant decrease in the expression of *rhodopsin* (*p* = 0.0008) was observed in 12-month-old *Asrgl1^mut/mut^* mice compared with age-matched wildtype mice. The *p* values < 0.01, and 0.001 are indicated with **, and ***, respectively.

***Morphology of cone Photoreceptors in the Asrgl1^mut/mut^ mice retina:*** The morphology of cones was evaluated by performing immunostaining with cone photoreceptor marker antibodies. Immunostaining of retinal sections from 3-, 9-, 13-, and 17-month-old *Asrgl1^mut/mut^* mice with short (OPN1SW) and medium (OPN1MW) wavelength cone opsin antibodies revealed a progressive loss of outer segments of both OPN1SW- and OPN1MW-expressing cones in the retinas of *Asrgl1^mut/mut^* mice with age (Figure 5A). However, the morphology of cones expressing both OPN1SW and OPN1MW was observed to be normal in wildtype mice (Figure 5A). Immunostaining with rhodopsin showed an apparent reduction in the thickness of OS length with age (Figure S2). Additional analysis is needed to quantify the extent of the decrease in OS length in *Asrgl1^mut/mut^* mice.

***Expression profile of the rod and cone photoreceptor marker genes:*** To study the integrity of photoreceptor layers, the expression of photoreceptor maker genes was analyzed. The levels of *Opn1sw*, *Opn1mw*, and *Rhodopsin* transcripts were significantly decreased at 3 months, and further decrease was observed at 12 months in *Asrgl1^mut/mut^* mutant mice retina compared with that of 12-month-old age-matched wildtype control mice. The expression of *Opn1sw* was significantly lower in 3- (*p* = 0.0044) and 12-month-old (*p* = 0.0001) *Asrgl1^mut/mut^* mice retina compared with that in 12-month-old wildtype control mice (Figure 5B). Similarly, a significant decrease in the expression of *Opn1mw* was detected in *Asrgl1^mut/mut^* mutant mice at 3 (*p* = 0.0004) and 12 months (*p* < 0.0001) compared with 12-month-old age-matched wildtype controls (Figure 5B). Furthermore, this decrease in the expression of *Opn1mw* was observed in *Asrgl1^mut/mu^*^t^ mutant mice as early as 3 months of age (*p* = 0.0004). Finally, the level of expression of *Rhodopsin* was also decreased (*p* = 0.0165) in *Asrgl1^mut/mut^* at 3 months and even more significantly (*p* = 0.0008) at 12 months compared with that in age-matched wildtype control mice.

**Figure 6:**
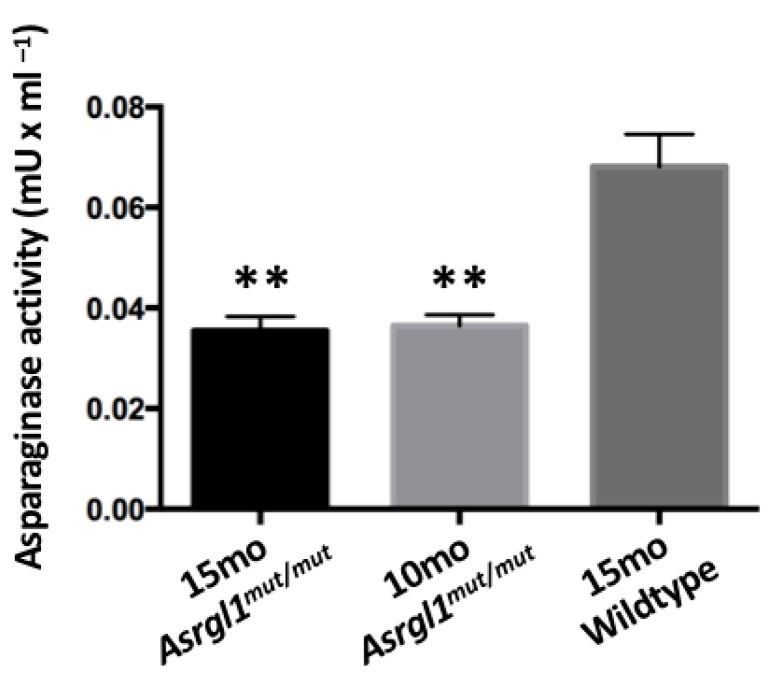
**Asparaginase activity in retinal tissue:** asparaginase activity detected in 10- and 15-month-old *Asrgl1^mut/mut^* mice retina was significantly lower (*p* < 0.0001) compared with the activity observed in 10-month-old wildtype mice. The *p* value < 0.01 is indicated with **.

***Asparaginase activity in Asrgl1^mut/mut^ mutant mouse:*** The asparaginase activity observed in the retinal lysates of 10- and 15-month-old *Asrgl1^mut/mut^* mice was significantly lower (*p* < 0.0001) compared with the activity in the retinal lysates of 10-month-old wildtype control mice (Figure 6).

## 4. Discussion

Patients with the G178R mutation in *ASRGL1*, which results in impaired asparaginase activity, show an onset of vision abnormalities in the first decade of life including decreased rod and cone response with progressive vision loss. Night blindness is reported to be among the first symptoms in these patients, suggesting rod photoreceptor abnormalities at early stages [11]. According to the findings, the rod and cone photoreceptors in the *Asrgl1* ablated mouse model developed progressive and significant structural and functional abnormalities beginning as early as 3 months of age (Figure 3). Specifically, a significant loss of s-opsin-expressing cones was observed in 3-month-old mice, while significant abnormalities in both m-opsin- and s-opsin-expressing cones were noted from 9-month-old mice (Figure 3 and Figure 5). Overall, the retinal phenotype of the *Asrgl1* ablated mouse model recapitulated the cone–rod dystrophy phenotype observed in patients with the G178R mutation in *ASRGL1*.

The sequence change introduced in *Asrgl1^mut/mut^* mice is predicted to result in a frameshift mutation leading to nonsense-mediated decay of the transcript (Figure 1). The minimal levels of the ASRGL1 transcript and protein (Figure 4) as well as the low levels of asparaginase activity observed in the retina of *Asrgl1* gene ablated mice (Figure 6) indicate loss of the transcript and, consequently, the enzymatic activity. Earlier in vitro studies revealed a significant loss of asparaginase activity due to the G178R mutation that was detected in patients with retinal degeneration and suggested that appropriate retinal structure and function are dependent on ASRGL1 activity [11]. The early onset and progressive rod and cone photoreceptor abnormalities observed in the *Asrgl1*^mut/mut^ mutant mouse model with minimal to undetectable levels of *Asrgl1* expression and asparaginase activity in the retina further support a critical role for ASRGL1 in the normal function of the retina.

The specific biological role of ASRGL1 and the impact of the loss of this protein in photoreceptors are unknown. The ASRGL1 protein has both L-asparaginase and β-aspartyl peptidase activities [11,12,17,18]. This protein is suggested to play a role in the production of L-aspartate, which acts as a neurotransmitter in the brain [12]. The expression of *ASRGL1* was reported in multiple tissues including adipose, brain, mammary glands, cervix, fallopian tube, lung, kidney, prostate, testes, cervix, and uterus (https://gtexportal.org/home/ accessed on 1 January 2022). Several studies demonstrated a strong association between high levels of ASRGL1 expression and tumorigenesis [12,19]. L-asparaginase is widely used in chemotherapy to treat adults and children with acute lymphoblastic leukemia [20,21]. ASRGL1 is widely expressed in non-ocular tissues, and while tissues other than the retina of the *Asrgl1* gene ablated mouse model have not been investigated in detail, a gross examination of the major organs of these mice did not reveal significant morphological abnormalities. Therefore, a significant decrease in the activity of *ASRGL1* or the expression of the *ASRGL1* transcript at minimal to undetectable levels may not result in major morphological consequential abnormalities in mice other than the retinal abnormalities that were evaluated in depth.

In a recent study, Zhou et al. reported that the deletion of exons 3 and 4 of *Asrgl1* led to late-onset photoreceptor abnormalities beginning from 8 months of age in a mouse model [22]. The retinal structure and expression of marker genes were normal up to 8 months without increased TUNEL-positive cells compared with controls. The ERG responses were slightly abnormal at 8 months, while they were reported to be normal at 6 months. A progressive and severe reduction in the outer nuclear layer was reported from 9 to 15 months. Overall, these mice were reported to have normal retinal structure and function up to 6 months with a progressive photoreceptor loss starting from 8 months of age. In contrast, the *Asrgl1^mut/mut^* mouse model with the homozygous c.578_579insAGAAA insertion developed photoreceptor abnormalities starting from 3 months of age including abnormal photopic ERG response and a significant decrease in the expression of rod and cone photoreceptor marker genes. The presence of abnormal and significantly short cone outer segments was observed at 9 months in *Asrgl1^mut/mut^* mice, whereas the *Asrgl1* KO mice reported by Zhou et al. developed similar changes in cone morphology at 12 months [22]. The major genotype difference between these models is the presence of the *Asrgl1* transcript without exons 3 and 4 in *Asrgl1* KO mice vs. the loss of the *Asrgl1* transcript and protein in the *Asrgl1^mut/mut^* mouse model. The impact of shorter *Asrgl1* transcript without exons 3 and 4 to the phenotype in *Asrgl1* KO mice is unknown. Comparison of the retinal changes between the *Asrgl1^mut/mut^* mouse model described in this study and the *Asrgl1* KO model reported by Zhou et al. revealed that the mice in both models developed progressive retinal degeneration involving both rod and cone photoreceptor loss [22], further supporting a key role for ASRGL1 in the retina and providing proof that a lack of ASRGL1 leads to retinal pathology.

The onset of retinal degeneration is observed to be earlier in the *Asrgl1^mut/mut^* mouse model at 3 months compared with the late-onset degeneration reported in the *Asrgl1* KO model at 8 months. However, the photoreceptor loss is much more severe in the *Asrgl1* KO mice involving the loss of both rods and cones by 15 months, whereas the *Asrgl1^mut/mut^* mice developed severe cone abnormalities by 3 months, while the ONL thickness appeared to be normal at 17 months, suggesting a less severe rod loss. The difference observed in the phenotype of these two mouse models, one (*Asrgl1^mut/mut^*) with predominant cone loss and mild rod abnormalities and the other (*Asrgl1* KO) with severe rod–cone degeneration despite the loss of the *Asrgl1* transcript in both, may be due to the contribution of the genetic background. Comparative analysis of these two models may provide an opportunity to understand the underlying cause of severe cone degeneration sparing rods in the *Asrgl1^mut/mut^* model even at older age.

Earlier studies on a zebrafish knockdown model injected with either start site morpholino (MO) or splice site MO of *asrgl1* did not reveal gross morphological abnormalities including lack of retinal abnormalities on 6 dpf [11]. However, the zebrafish model injected with human mutant hASRGL1 mRNA showed a loss of blue-opsin-expressing cones. Evaluation of zebrafish *Asrgl1* knockout and mutant knock-in models developed by editing the genome is needed to compare the impact of *Asrgl1* gene ablation on rod and cone photoreceptors in the retina of zebrafish.

While the *Asrgl1* gene ablated mouse models establish the involvement of this gene in retinal degeneration, the specific role of ASRGL1 and the mechanism underlying the progressive retinal degeneration due to the loss of ASRGL1 expression and/or activity are unknown. This novel *Asrgl1^mut/mut^* mouse will serve as a model to understand the role of ASRGL1 in the retina. Furthermore, this novel *Asrgl1^mut/mut^* mouse recapitulating the phenotype of patients with G178R ASRGL1 mutation will serve as a model for preclinical evaluation of therapeutic strategies to treat patients with mutations specifically in ASRGL1 and cone–rod dystrophy in general [11]. Additionally, these mice may also serve as a model to study the role of ASRGL1 in tumorigenesis and related pathologies.

## Figures and Tables

**Table 1:** Primer sequences for the *Asrgl1^mut/mut^* mice genotyping.

Primer ID	Sequence	Length	Tm	GC%	Amplicon
**1**. mAsrgl1 wt F	AAGCCAAGTTTCCTCTGAAGTC	22	60.3	45.5	328 bp
**2**. mAsrgl1 mut F	AAGCCAAGTTTCCTCTGGTTCT	22	60.3	45.5	333 bp
**3**. mAsrgl1 uni R	ACCAAGCCAACCTCAGACTC	20	60.5	55.0	

Footnote: Primers are shown 5′ to 3′.

**Table 2:** Primer details for the *Asrgl1^mut/mut^* mice genotyping.

Sample	Set A (Wildtype Allele Specific)	Set B (Mutant Allele Specific)
Wildtype **(*Asrgl1^−/−^*)**	328 bp product	None
Homozygous **(*Asrgl1^mut/mut^*)**	None	333 bp product
Heterozygous **(*Asrgl1^mut/−^*)**	328 bp product	333 bp product

## Data Availability

Not applicable.

## Supplementary Materials

The following supporting information can be downloaded at: https://www.mdpi.com/article/10.3390/genes13081461/s1, Figure S1: Fundus images of *Asrgl1^mut/mut^* mice model: representative fundus images obtained by autofluorescent scanning laser ophthalmoscopy (AF-SLO) imaging taken at 55° angle lens from both wildtype and *Asrgl1^mut/mut^* mice at 4 and 12 months; Figure S2: Rhodopsin staining of *Asrgl1^mut/mut^* mice model: the thickness of OS length appears to show an apparent reduction in retinal sections of 3- and 13-month-old *Asrgl1^mut/mut^* mice immunostained with Rho (stained in green). RPE: retinal pigment epithelium; OS: outer segments; ONL: outer nuclear layer; OPL: outer plexiform layer; INL: inner nuclear layer; IPL: inner plexiform layer; GCL: ganglion cell layer.
