# Supplementary material for: A Mouse Model with Ablated Asparaginase and Isoaspartyl Peptidase 1 (Asrgl1) Develops Early Onset Retinal Degeneration (RD) Recapitulating the Human Phenotype
Source: Genes (Basel). 2022 Aug 17;13(8):1461. doi: 10.3390/genes13081461 (PMC9408336; doi:10.3390/genes13081461)
Supplement: Supplementary file 1 [file genes-13-01461-s001.zip › genes-1818836-supplementary.pdf]

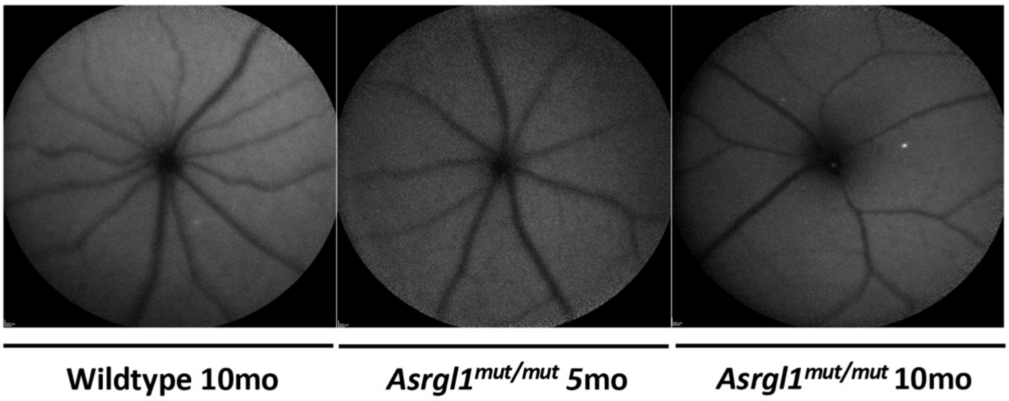

Figure S1. Fundus Images of *Asrgl1$^{mut/mut}$* mice model: Representative fundus images obtained by autofluorescent scanning laser ophthalmoscopy (AF-SLO) imaging taken at 55° angle lens from both wildtype and *Asrgl1$^{mut/mut}$* mice at 4 months and 12 months.

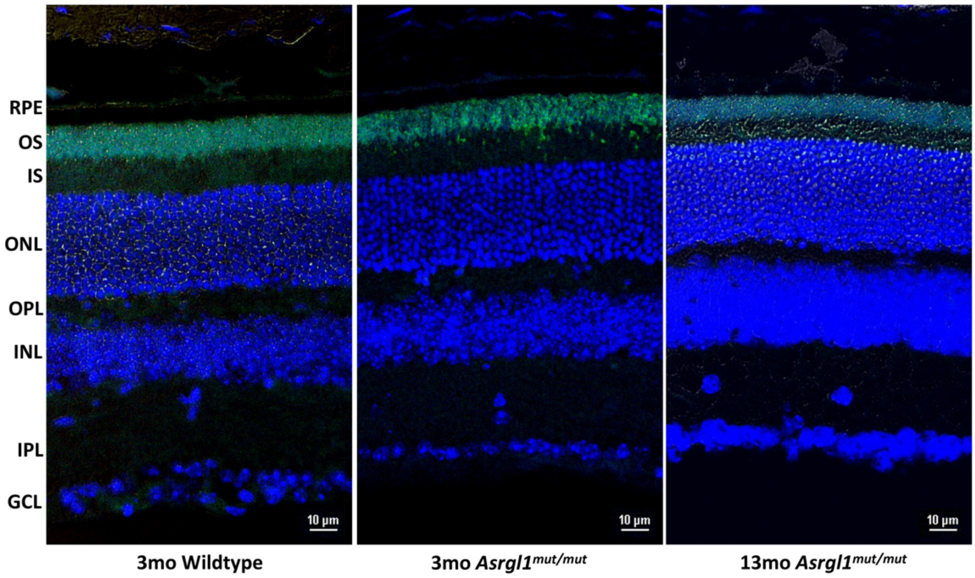

Figure S2: Rhodopsin staining of *Asrgl1$^{mut/mut}$* mice model: The thickness of OS length appears to be reduced in retinal sections of 3 and 13 month old *Asrgl1$^{mut/mut}$* mice immunostained with Rho (stained in green) show apparent. RPE: Retinal pigment epithelium; OS: Outer Segments; ONL: Outer Nuclear Layer; OPL: Outer Plexiform Layer; INL: Inner nuclear layer; IPL: Inner Plexiform Layer; GCL: Ganglion Cell Layer.
